# Digital soil mapping including additional point sampling in Posses ecosystem services pilot watershed, southeastern Brazil

**DOI:** 10.1038/s41598-019-50376-w

**Published:** 2019-09-24

**Authors:** Bárbara Pereira Christofaro Silva, Marx Leandro Naves Silva, Fabio Arnaldo Pomar Avalos, Michele Duarte de Menezes, Nilton Curi

**Affiliations:** Departamento de Ciência do Solo, Universidade Federal de Lavras UFLA, Av. Doutor Sylvio Menicucci, 1001, Kennedy, Lavras, MG Brazil

**Keywords:** Environmental sciences, Environmental chemistry

## Abstract

This study aimed to evaluate the performance of three spatial association models used in digital soil mapping and the effects of additional point sampling in a steep-slope watershed (1,200 ha). A soil survey was carried out and 74 soil profiles were analyzed. The tested models were: Multinomial logistic regression (MLR), C5 decision tree (C5-DT) and Random forest (RF). In order to reduce the effects of an imbalanced dataset on the accuracy of the tested models, additional sampling retrieved by photointerpretation was necessary. Accuracy assessment was based on aggregated data from a proportional 5-fold cross-validation procedure. Extrapolation assessment was based on the multivariate environmental similarity surface (MESS). The RF model including additional sampling (RF*) showed the best performance among the tested models (overall accuracy = 49%, kappa index = 0.33). The RF* allowed to link soil mapping units (SMU) and, in the case of less-common soil classes in the watershed, to set specific conditions of occurrence on the space of terrain-attributes. MESS analysis showed reliable outputs for 82.5% of the watershed. SMU distribution across the watershed was: Typic Rhodudult (56%), Typic Hapludult* (13%), Typic Dystrudept (10%), Typic Endoaquent + Fluventic Dystrudept (10%), Typic Hapludult (9.5%) and Rhodic Hapludox + Typic Hapludox (2%).

## Introduction

Knowledge of the geographic distribution of soils allows the assessment of environment-soil relationships at the landscape level^1^. Such information is essential for agronomic assessment, soil and water management and land use planning^1–3^. Digital soil mapping (DSM) techniques are supported by a well-known and widely accepted model in soil science: the factors of soil formation. Early efforts to bring a quantitative solution to this model could be traced back to the work of Jenny^4^. Currently, with the development of geographic information technology and data processing, a comprehensive digital framework could be applied for the production of digital soil maps^1,5,6^.

The *scorpan* model, as formalized by McBratney *et al*.^5^, serves as a route for the production of digital soil maps. It establishes a quantitative model for spatial association between soil forming factors, evaluated as covariate maps, and the occurrence of soil classes or properties. However, since the spatial association complexity is highly variable among landscapes, there is still no consensus or a pre-established function or operational model (spatial inference models) that could relate soilscape features and the occurrence of soil classes. Hence, model selection still represents a challenge in DSM studies.

Spatial inference models could be divided into data-driven (pedometric approach) and knowledge-driven approaches^7^. The pedometric approach (automatic and quantitative, includes statistics, geostatistics, machine learning, and data mining) gives a predictive accuracy that is generally related to a dense sampling scheme^8^. In the knowledge-driven approach, the knowledge of pedologists is incorporated into spatial prediction^9^ and the soil maps are viewed as a representation of the pedologist’s understanding of the soils^10^. Such tacit knowledge is mostly based on a paradigm of conceptual soil-landscape model, under the hypothesis that the location and distribution of soils in the landscape is predictable^11^. Thus, the use of spatial inference models as fuzzy logics^12^, or bayesian inference^13^, to name a few, take advantage of such concepts in predictive maps.

While current algorithms applied in DSM have shown acceptable performances^14–16^, there is still a concern related to the occurrence of less-common soil classes across different landscapes. These soil classes can have significant effects on the process of model fitting, model selection, and output accuracies^17^. To overcome this issue, different strategies could be adopted, e.g. feature-space oversampling^18^ or geographic space oversampling^19^. Although these data-driven strategies have proved to be useful in specific circumstances^17^, some pedological aspects related to the occurrence of certain soil classes remain unsolved. Although pedometric and knowledge-driven approaches differ in philosophy and technical emphasis^7^, they are not mutually exclusive^20^. In this sense, pedologists’ knowledge about the study area (mental model) could also be incorporated into the digital mapping process, by identifying characteristic sites in aerial imagery associated to soil profiles previously surveyed^21^, which might be useful to account for specific conditions of less-common soil classes. In this way, a qualitative soil-landscape model would be translated into quantitative predictions supported by the spatial association between soil classes occurrence and environmental covariates^22^.

Different techniques of DSM have been widely assessed in studies in tropical soils^23–27^. However, mountainous and complex relief areas still present challenges to any approach to soil mapping, due to the complex and scale-dependent interactions among soil forming factors and the cost-effort associated to survey inaccessible areas. Therefore, this study aimed to evaluate the performance of three algorithms used in digital soil mapping and the effects of additional sampling in the presence of less-common soil classes in a steep-slope watershed.

## Material and Methods

### Study area

**Figure 1:**
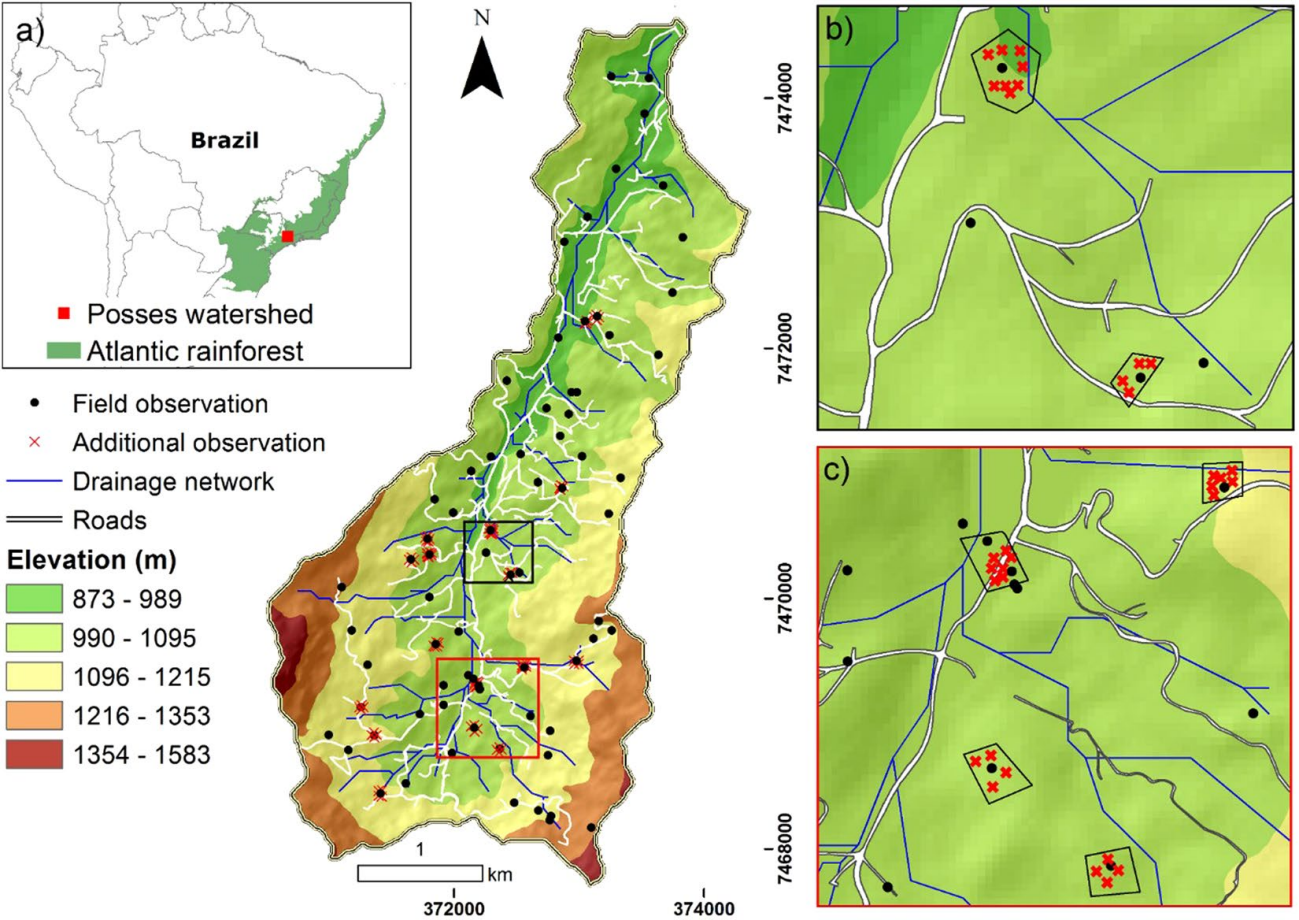
(**a**) Location of the study area and the soil survey sampling sites and their associated additional observations. (**b**,**c**) Detail of the field and additional observations clusters.

This work was carried out at the Posses watershed, southeastern Brazil (Fig. 1). This watershed covers an area of 1,200 ha, has an altitude range of 945 to 1,435 m, and a relief dominated by steep (>20%) to very steep slopes (>60%). According to Köppen’s climate classification system, it corresponds to the Cfb class, i.e., mesothermic with no dry season and a warm summer^28^. The average annual temperature is 18 °C, the hottest and coldest months have average temperatures of 25.6 °C and 13.1 °C, respectively, and the average annual precipitation is 1,447 mm. The predominant parental materials are alkaline granite and monzonite^29^.

This water-production watershed is a pilot site for the “Conservador das Águas” project, which stands, since 2006, as a pioneering initiative to establish a payment system for ecosystem services in Brazil^30^. Among the objectives of this project is the promotion of the sustainability of water resources for the Cantareira System, which is the major water supply system for the metropolitan region of São Paulo city.

### Soil survey

The soil survey was carried out in accordance with Brazilian technical requirements of a semi-detailed soil survey^31^. For this study area, the requirements are: (a) mapping units composed by a single taxonomic unit; (b) a total of 16 samples/hectare, and (c) free-way sampling scheme, supported by the experience of the pedologists along with cartographic products (mainly digital terrain models). The later was used in order to capture the soil-landscape relationships and promote the sampling in different landforms (resulted from the interaction of soil forming factos). It has been considered as the traditional basis of variability capturing in soil surveys, historically based on dual well-accepted paradigms: soil forming factors and soil–landscape relationships (origin in the soil factor equation outlined by Dokuchaeiv^32^ and Hilgard^33^.

Every point was visited by means of GPS/GNSS navigation and the coordinates of sampled sites were registered again with a nominal accuracy of ~10 m; such location accuracy allowed a spatial resolution mapping of 20 m^34^. At each point soil profiles morphological description and collection of samples from horizons were carried out as support for soil taxonomic classification^35^.

### Soil mapping units

The soils surveyed in the study area were classified according to Soil Survey Staff^35^ in: Typic Dystrudept, Typic Humudept, Typic Endoaquent, Fluventic Dystrudept, Rhodic Hapludox, Typic Hapludox, Typic Hapludult (yellow), Typic Rhodudult, Rhodic Kandiudult and Typic Hapludult* (red-yellow) (Table 1), which correspond to Haplic Cambisol, Humic Cambisol, Haplic Gleysol, Fluvic Cambisol, Red-Latosol, Red-Yellow Latosol, Yellow Argisol, Red Argisol, Red Nitosol, and Red-Yellow Argisol in the Brazilian Soil Classification System^36^, respectively.

**Table 1:** Distribution of pedons per soil mapping unit in Posses watershed.

Soil mapping unit	Inclusion	FO	AO
Typic Dystrudept	Typic Humudept	6	19
Typic Endoaquent + Fluventic Dystrudept	—	4	14
Rhodic Hapludox + Typic Hapludox	—	3	20
Typic Hapludult (yellow)	—	10	16
Typic Rhodudult	Rhodic Kandiudult	36	0
Typic Hapludult* (red-yellow)	—	15	12

FO = field observation; AO = additional observations.

### Additional observations

**Figure 2:**
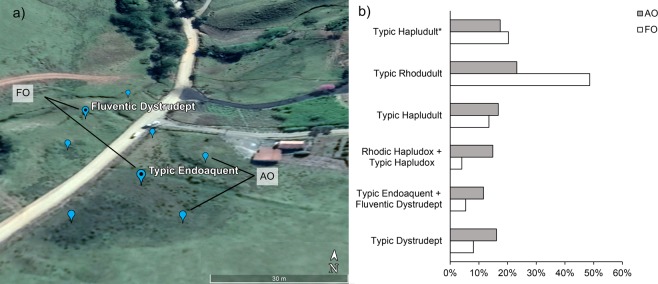
(**a**) Allocation of additional observations (AO) to field observations (FO) in Google Earth for the soil mapping unit (SMU) Typic Endoaquent + Fluventic Dystrudept. Source: Google Earth Pro 7.3, https://www.google.com.br/earth/download/gep/agree.html. (**b**) Original and final proportions of the training datasets by SMU.

Although technical recomendations of soil survey were answered, for an proper algorithm performance, additional observations were created (AO), since the increasing of in-field sampling points is economical unfeasible. In addition, the proportion of sampling in less common landscapes (small proportions) is consequently smaller. In this sence, once the dominant soil mapping unit (SMU) was the Typic Rhodudult taxon (49% of the surveyed locations), this fact resulted in a highly imbalanced dataset (Table 1). To overcome this issue, AO were allocated for the less common soil mapping units by means of our ‘expert knowledge’ and photointerpretation from high-resolution imagery in Google Earth (Fig. 2). From the paradigm that support traditional soil surveys, according to Hudson^37^ the understanding of soil-landscape paradigm leads to soil-landscape units concepts, which consists of natural terrains resulted from the interaction of soil forming factors. Generally, the more similar two landscape units are, the more similar their associated soil tend to be, and vice-versa. Such concepts guided the choose of AO points.

The procedure of automatic allocation of AO to increase the sample size for model training has been used in some DSM applications with variable improvements in accuracy^27^ since it depends on the degree of spatial autocorrelation among soil classes, which is specific to different landscapes. Consequently, an automatic approach should be applied prudently. Therefore, we adopted a ‘supervised’ approach based on the following criteria: AO points were prevented from occurring within 20 m of one another (mapping resolution) and within 20 m of previously surveyed pedon (field observation, FO) (Fig. 2a). The AO points were also constrained as to not exceed an imbalance greater than a proportion of 2 (dominant) to 1 (less common) (Fig. 2b). Photointerpretation is a conventional technique used in soil mapping that could be included in DSM operations to increase the representation of the feature-space of environmental covariates by attaching additional observations to the training data^38,39^.

### Soil covariates

The spatial prediction framework was based on the approach proposed by McBratney *et al*.^5^, which is an updated and spatially-explicit implementation of the early concepts of Dokuchaev’s soil forming factors and Jenny’s quantitative interpretation of them^4^. This technique assumes that a soil property or class is a function of a spatial representation of other soil property (s) or a soil-forming factor, namely: climate (c), organisms and vegetation (o), topography (r), parent material (p), time (a), and geographic location (n).

Climate (c) and parent material (p) proxies are not available at the scale of analysis of this study (10^1^ km), and the coarse resolution of current information does not represent spatial variation that could be used for digital mapping. Therefore, only the available maps of relief (r) and organisms and vegetation (o) factors were assessed.

The relief factor (r) proxy was a high-resolution ALOS PALSAR digital elevation model (DEM)^40^ accessed through www.asf.alaska.edu. The DEM’s spatial resolution was 12.5 m and was upscaled to the earlier mentioned spatial resolution mapping of 20 m by bilinear interpolation. Subsequently, it was hydrologically corrected, and a total of 28 terrain attributes (see Supplementary Table S1), that are commonly tested for digital soil mapping, were derived.

Sentinel 2 imagery data (top of the atmosphere reflectance), downloaded from scihub.copernicus.eu, were assessed as the organisms and vegetation factor (o). In addition to reflectance data, normalized difference vegetation index (NDVI)^41,42^ and normalized difference water index (NDWI)^43^ were calculated and evaluated (Supplementary Table S1). Processing of *o* and *r* factors was carried out in SAGA GIS software^44^ and the model analysis was run in R^45^.

### Model selection and soil map production

Aiming to fit the best spatial association model that relates soil-forming factor proxies and the occurrence of soil mapping units, three models were tested: Multinomial Logistic Regression (MLR), C5 Decision Tree (C5-DT), and Random Forest (RF).

The MLR analysis allows to model categorical responses by fitting linear combinations of a set of covariates (predictors) to the natural logarithm of the odds of every level of a response variable (logit transformation)^46,47^. MLR has been widely applied and tested for classification and mapping of soil features^15,48^. Therefore, the set of soil covariates were evaluated as predictors of the probability of occurrence of the soil mapping units. MLR analysis has been implemented following the best subset procedure that involved testing every combination of the soil covariates for the prediction of the soil mapping units. The best MLR model was selected on the basis of the Bayesian Information Criterion (BIC), being that the model with the lowest BIC was selected.

Classification and Regression Trees (CART) are non-parametric and rule-based models that have a tree-structure and are based on the mining of relationships between a target variable and the feature-space of a set of covariates^49^. The C5-DT algorithm (an updated version of C4.5^50^), in a similar way to other CART methods, uses a recursive process that relies on the partition of the feature-space and the separation of the observed classes until a stopping criterion is met (e.g. Gini index, Shannon entropy^49^). The performance of C5-DT in soil mapping has been proven as efficient^16^, notably when non-linear and complex soilscape relationships are intended to be modeled. A C5-DT model was fitted with the default parameters in the C5.0 R’s package^50^.

The RF algorithm is also a CART method that records remarkable performances in DSM^14,15^; it differentiates from other CART by being an ensemble and bootstrapping method^51,52^. Since the most sensitive parameter in RF fitting is *mtry*, i.e., the number of candidate covariates tested on each split^52^, a test was run to find the *mtry* value that produced the lesser out-of-bag classification error. Subsequently, in order to facilitate interpretation and eliminate redundant covariates, a subset of the most important covariates, as calculated by the mean decrease in accuracy in the RF output^51,52^, was selected; then, from this subset, high correlated covariates(Spearman rank correlation >0.85) were removed for subsequent analysis.

Model selection criteria and the effects of additional sampling were evaluated on accuracy statistics (global accuracy, kappa index and producer’s and user’s accuracies) aggregated by the mean values from a repeated 5-fold cross-validation procedure, which is recommended for imbalanced and relatively small samples^53^; every fold in this operation precluded the inclusion of the cluster formed by the FO and its associated AO (Fig. 1b,c), being that accuracy statistics estimates are affected by spatial autocorrelation^19^. Subsequently, after 100 repetitions, the model with best accuracy metrics was applied for the production of the digital soil mapping and further soilscape analysis. In order to test the performace of this approach, a comparison with a repeated 5-fold cross-validation procedure using only field observations was performed.

### Multivariate environmental similarity surface (MESS)

Map quality related to the level of representation of the feature-space by the point-samples (level of extrapolation) was evaluated by calculating the Multivariate Environmental Similarity Surface (MESS), according to Elith *et al*.^54^ (Eq. 1).

1 $$P({V}_{i})=\{\begin{array}{ll}\frac{({p}_{i}-\,mi{n}_{i})}{(ma{x}_{i}-\,mi{n}_{i})}\,\times 100, & {f}_{i}=0\\ 2\times {f}_{i}, & 0 < {f}_{i}\le 50\\ 2\times (100-\,{f}_{i}), & 50\le {f}_{i} < 100\\ \frac{(ma{x}_{i}-\,{p}_{i})}{(ma{x}_{i}-mi{n}_{i})}\,\times 100, & {f}_{i}=100\end{array}$$

where: P (V_i_) is the multivariate similarity (MES) of a point; min_i_ = minimum value of the covariate Vi over the reference point set, max_i_ = maximum value of variable Vi over the reference point set; p_i = _value of the covariate V_i_ at point P; f_i_ = percent of reference points whose value of covariate V_i_ is smaller than p_i_. The MES of P is the minimum of its similarity with respect to each covariate.

MES surface is produced by applying Eq. 1 to every single pixel in the model output. This method quantifies the similarity between the training samples and the selected covariates; values less than zero indicate prediction locations, both in feature and geographic spaces, that have not been accounted for by the training samples. By doing this, it enables to represent spatially the level of extrapolation related to the sampling pattern of the study area. This approach has been used widely in species distribution modeling^54,55^ but its use is still scarce in DSM^14^. The evaluated feature-space was that made up by the selected covariates and the training samples of the best model.

**Figure 3:**
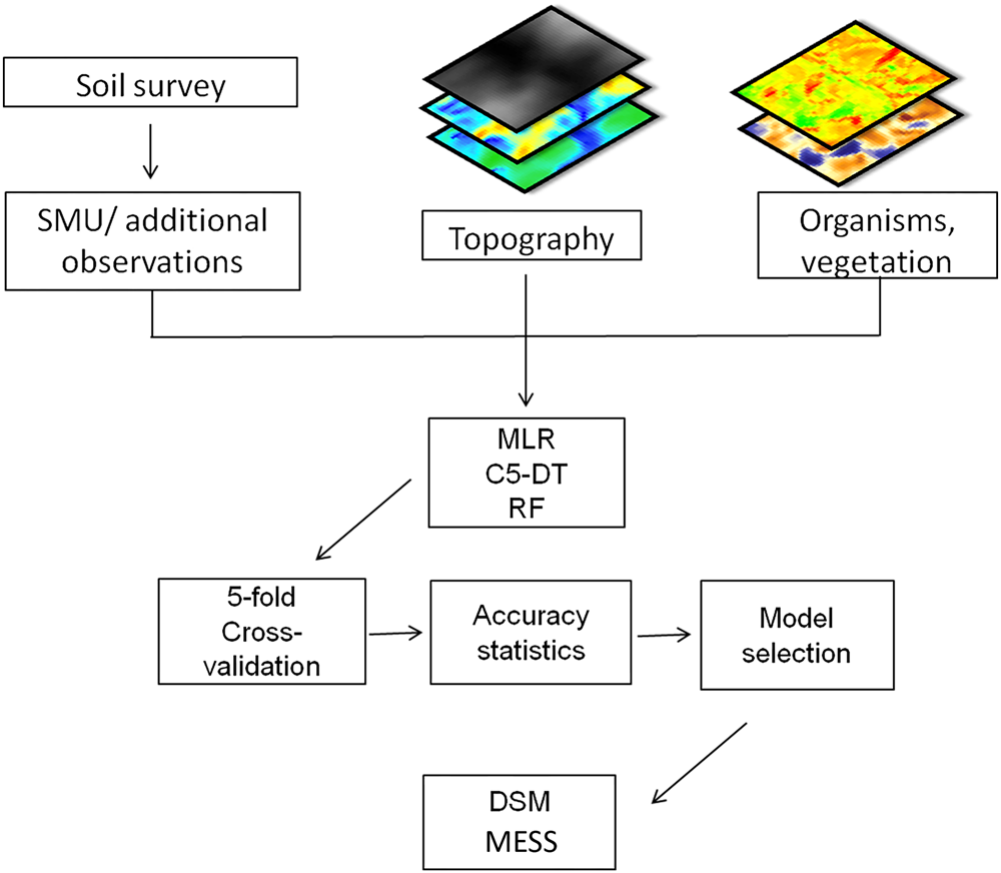
Soil map production flow chart. MLR: multinomial logistic regression, C5-DT: C5 decision tree, RF: random forest, DSM: digital soil map, MESS: multivariate environmental similarity surface.

The flow chart of the soil map production is shown in Fig. 3.

## Results and Discussion

### Accuracy assessment and model performance

**Figure 4:**
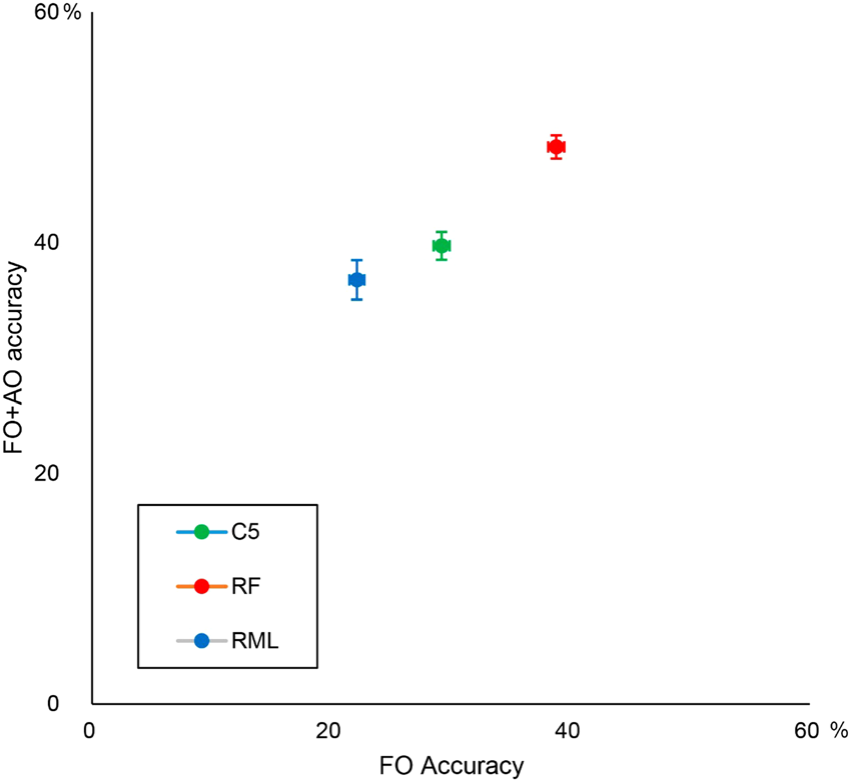
Mean overall accuracy of the tested models and the effects of additional observations (AO accuracy) in relation to field observations (FO accuracy). Bars are standard errors from 5-fold cross-validation after 100 repetitions. C5-DT: C5 decision-tree, RF: random forest, MLR: multinomial logistic regression.

The performance of the models to predict the soil mapping units registered in the Posses watershed is summarized in Table 2. Additional point sampling improved the accuracy of all models (Fig. 4).

**Table 2:** Accuracy statistics from repeated 5-fold cross-validation.

Subgroups	C5*	RF*	MLR*	C5^c^	RF^d^	MLR^e^
	Producer’s accuracy					
Typic Dystrudept	44	50	44	14	8	13
Typic Endoaquent + Fluventic Dystrudept	43	53	71	21	16	36
Rhodic Hapludox + Typic Hapludox	35	46	41	0	0	5
Typic Hapludult (yellow)	42	49	39	18	24	21
Typic Rhodudult	34	41	24	43	66	29
Typic Hapludult (red-yellow)	31	39	19	22	18	14
	User’s accuracy					
Typic Dystrudept	34	45	43	20	0	17
Typic Endoaquent + Fluventic Dystrudept	37	37	57	10	0	24
Rhodic Hapludox + Typic Hapludox	32	42	33	0	0	5
Typic Hapludult (yellow)	39	49	30	18	22	12
Typic Rhodudult	42	52	32	43	50	39
Typic Hapludult (red-yellow)	31	37	22	22	23	13
OA%^a^	40	49	37	29	39	22
SE%^b^	1.2	1	1.7	0.8	0.7	0.62
Kappa	0.22	0.33	0.20	0.04	0.07	−0.02

^a^Overall accuracy.

^b^Standard error.

^c^C5 Decision Tree.

^d^Random Forest.

^e^Multinomial logistic regression.

*Model including additional point sampling.

The models without additional observations were rarely able to predict less-common soils. This finding is consistent with data presented in other works (e.g. Barthold *et al*.^56^; Brungard *et al*.^17^; Jafari *et al*.^57^) and it is related to soil classes with very limited presence in the study area, in comparison with the dominating soil classes that are better represented in geographic and feature spaces.

Additional point sampling by photointerpretation enabled to capture specific conditions of occurrence of less-common soils and, in consequence, it was showed a substantial improvement in classification accuracy of the minority soil classes. This knowledge-driven technique could be regarded as an alternative to the data-driven approach and has already been integrated into predictive modeling with remarkable improvements in accuracy^26,46,58,59^. Nevertheless, there is still a need for a measure of their quality, as it depends only on the expertise of the photo interpreter, which restricts its application.

Accuracy statistics from the repeated 5-fold cross-validation demonstrates that the RF* model consistently outperformed the other models (overall accuracy = 49%, kappa index = 0.33), while MLR had the lowest overall accuracy and kappa index (Table 2 and Fig. 4). For the purposes of digital soil mapping, it has been frequently observed that complex models, such as RF, are better classifiers than generalized linear models such as MLR^14,17,60^. With regards to the accuracy rank in Table 2 (RF > C5-DT > MLR), it seems that the most sophisticated model fitted more complex relationships, as suggested by Heung *et al*.^60^. Since MLR is a generalization of the logistic regression, it is highly dependent on the presumed sigmoidal model for class discrimination^46^, which makes it the less flexible model. The C5-DT is a data-driven and non-parametric discrimination model, and, considering its single tree structure, it tends to show worse performances when compared to more sophisticated models. RF is also a data-driven and non-parametric model; however, its bootstrapping and bagging features are more efficient in revealing consistent and complex patterns^60^, especially when dealing with complex spatial associations, as occurred in this watershed, helping to explain its performance as the most accurate model.

The spatial association model obtained from RF* had also the highest producer’s and user’s accuracies (Table 2). In order to support the interpretation of the RF* output, we applied the ‘forest floor’ method^61^, which enables to visualization of the link between feature and prediction spaces, which we refer to as occurrence signatures.

### Analysis of covariates

**Figure 5:**
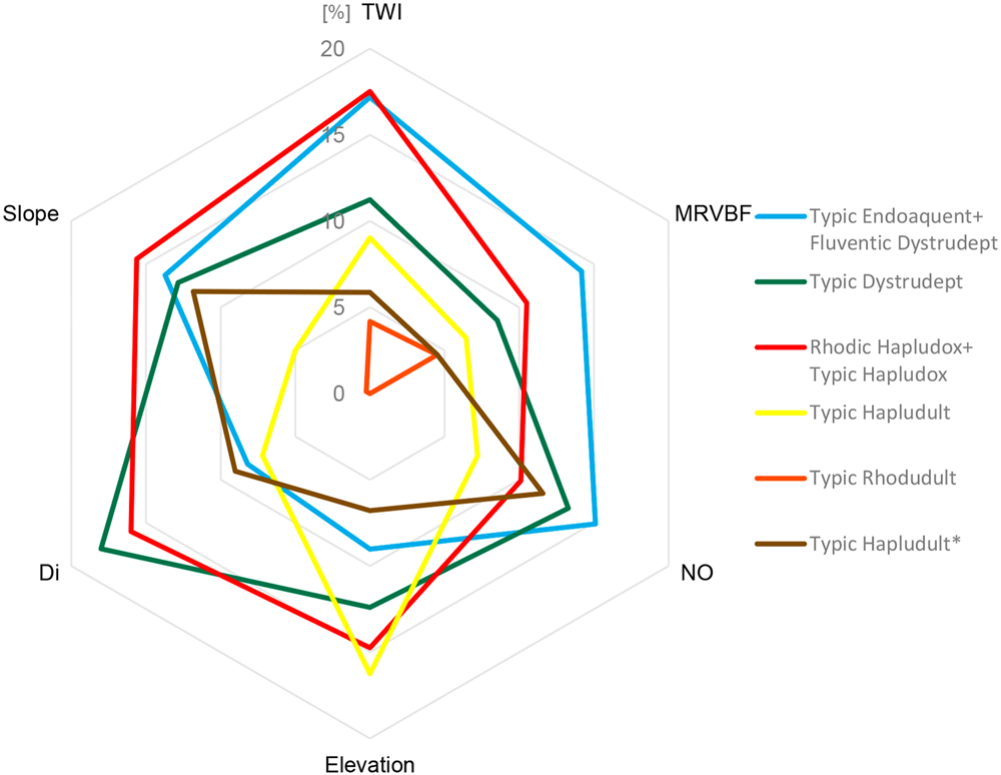
Importance of selected covariates by soil mapping unit. TWI: topographic wetness index; DI: direct insolation; NO: negative openness; MRVBF: multi-resolution index of valley bottom flatness.

The most important and the less correlated covariates, as determined by the mean decrease in accuracy (MDA) in the RF* model (MDA < 15%, see Supplementary Fig. S3) and the correlation criterion (ρ ≤ 0.85, see Supplementary Fig. S2), are presented in Fig. 5.

The topography proxy (r), as demonstrated by the mean decrease in accuracy (variable importance rank) in the RF* output, correlation analysis, and classification accuracies, had the best set of predictors, remarkably: SAGA topographic wetness index (TWI), slope, elevation, direct insolation (DI) and the multiresolution index of valley bottom flatness (MRVBF) (Fig. 5); while the vegetation and organisms (o) covariates showed very low importance (Supplementary Fig. S3) and were, consequently, removed from the analysis.

Regarding the global discrimination of the soil mapping units, the topographic wetness index (TWI) registered the highest importance score (Fig. 5). This terrain attribute is a substitute measure of water flux in the landscape, since it shows the tendency of a site to be water saturated and the possible drainage systems of a watershed^62^. TWI has often been reported as a potential predictor in digital soil mapping^56,63–66^.

**Figure 6:**
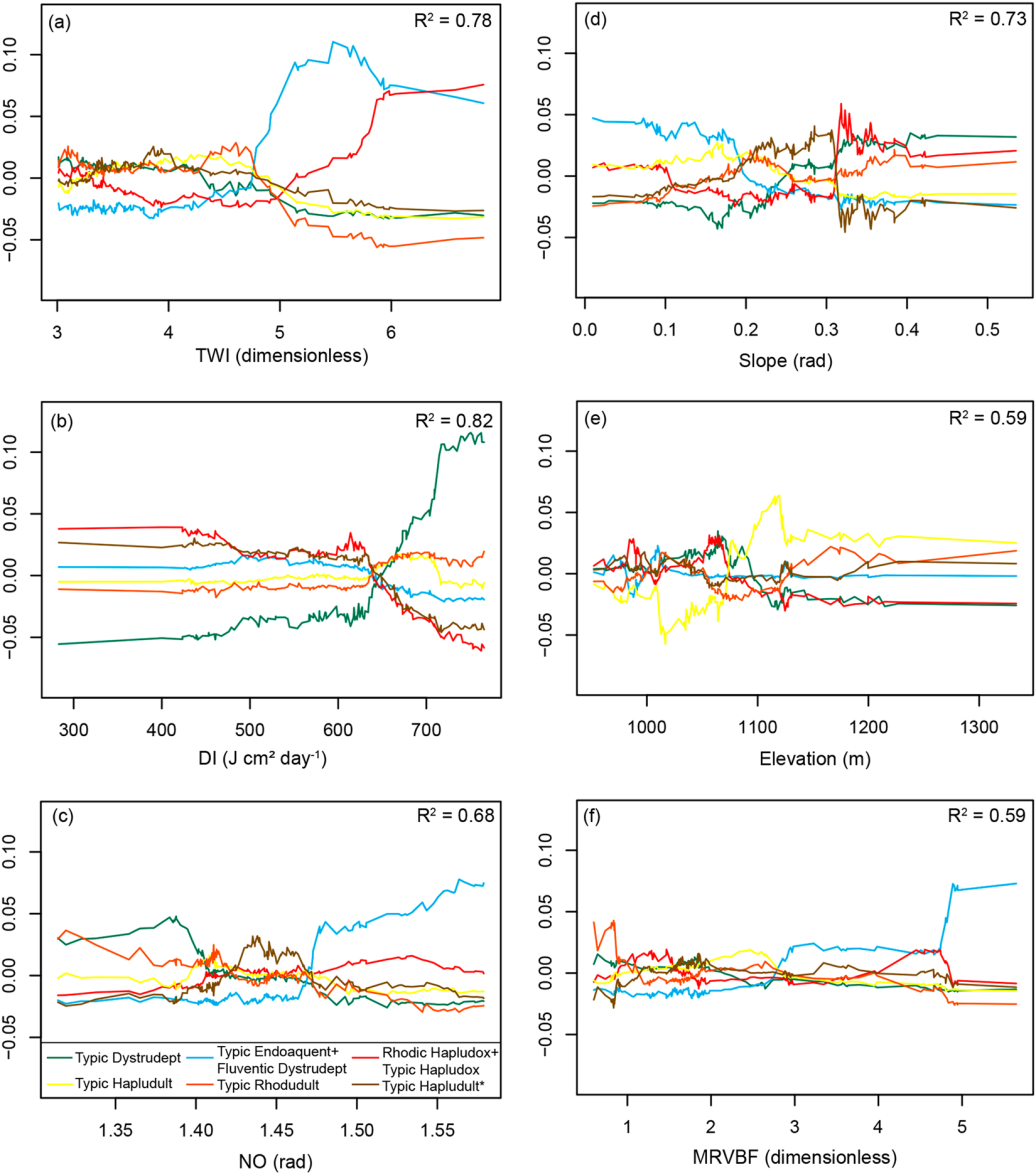
Occurrence signatures of soil mapping units projected on the feature-space of selected covariates. X-axis: covariate value, Y-axis: change of predicted class probability. R-squared values indicate goodness-of-fit between feature and prediction space projections (solid curves). TWI: topographic wetness index; DI: direct insolation; NO: negative openness; MRVBF: multiresolution index of valley bottom flatness.

The TWI stands out as a specific predictor for the Typic Endoaquent + Fluventic Dystrudept mapping unit. Its influence on the discrimination of this soil mapping unit is verifiable for values above 4.5, where an increase of 10% in probability of occurrence is observed (Fig. 6a). The occurrence of Rhodic Hapludox + Typic Hapludox mapping unit is also influenced by TWI. These two soils classes could not be distinguished in the final map because they occur randomly distributed in the same landscape position. Silva *et al*.^66^ also reported the influence of TWI on the distinction of Oxisols in Brazil. Higher TWI values (>5, i.e., poorly drained areas), supported also better discrimination among the Typic Rhodudult, Typic Endoaquent + Fluventic Dystrudept, and Rhodic Hapludox + Typic Hapludox mapping units (Fig. 6a).

The slope was the next important covariate for global classification. Flatter slopes are associated with a higher probability of occurrence of Typic Endoaquent + Fluventic Dystrudept mapping unit (Fig. 6d), which is expected to occur in flat slopes along with high values of TWI^48^. These two soil classes could not be separated in the final map due to their intricate occurrence pattern in the watershed landscape. Slope values around 30 radians influenced the discrimination between Typic Dystrudept and Rhodic Hapludox + Typic Hapludox (Fig. 6d). The slope covariate also registered the highest importance regarding Typic Hapludult*, where slope values above 30 radians showed an inflection point in the occurrence signature of this soil mapping unit (Fig. 6d).

Direct insolation (DI), ranked as third in importance for global classification, represents a pixel-based calculation for solar radiation^67^. This covariate has received little investigation in tropical conditions^68,69^. The DI covariate was a remarkable predictor for Typic Dystrudept. Values above 600 J cm² day^−1^ were associated with an increase in the probability of occurrence of this mapping unit, while lower values were associated to the occurrence of the Rhodic Hapludox + Typic Hapludox and Typic Hapludult* (Fig. 6b). The elevation covariate was relevant for Typic Hapludult distinction; Fig. 6e presents the occurrence signature of this soil mapping unit, where a distinctive pattern is observed between 1000 and 1200 m range.

While the RF* model satisfactorily discriminated the soil mapping units, a certain degree of mixture is still present in model outputs (Fig. 6). This could be an effect of the dominant soil and its wide range of occurrence conditions in the watershed. On the other hand, mapping units with a lesser degree of ‘impurity’ are those related to specific conditions across the landscape (Typic Dystrudept and Typic Endoaquent + Fluventic Dystrudept).

The level of detail in the topographic data analyzed, both spatial and informative, allowed the identification of specific relief-soil interactions. Topography, as a surficial hydrologic driver, could also reflect other soil-forming factors that have a larger scale domain, by modifying locally the effects of bioclimate and parent material^6^.

### Multivariate environmental similarity surface (MESS)

**Figure 7:**
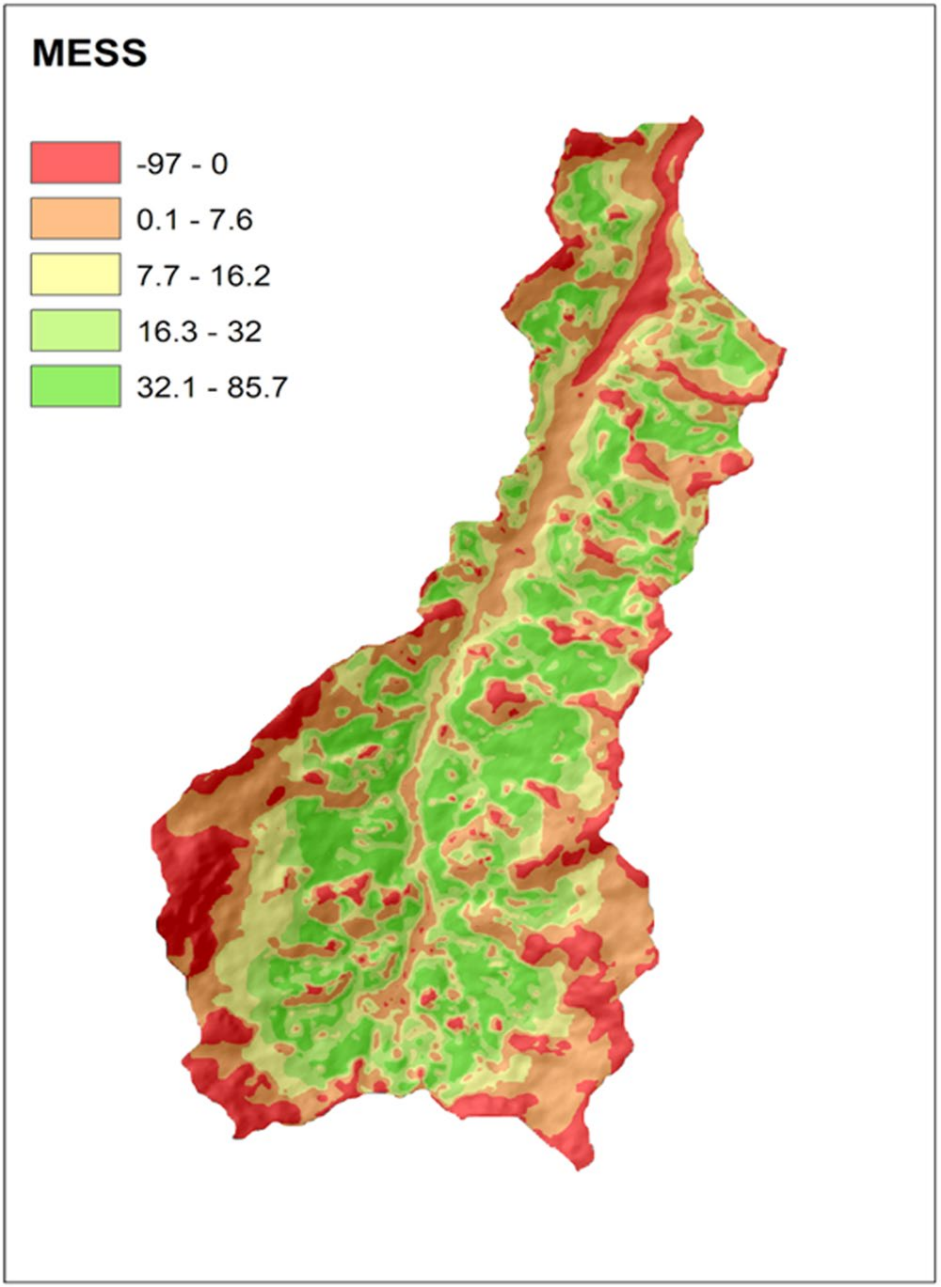
Multivariate environmental similarity surface (MESS) of the chosen model (random forest with additional observations), extrapolation level increase along with negative values.

In Fig. 7 it is possible to see the spatial distribution of the level of representation of the model prediction by the training data. The MESS measures the similarity of any given point to a reference set of points, with respect to the chosen predictor variables in the RF* model. Negative values indicate the presence of at least one variable out of reach of the reference points in feature-space, while positive values indicate greater similarity to the set of reference points. The higher the score, the more common the point is and more reliable is the prediction^54^. This feature enables to interpret the MESS as a quality measure of the sample configuration. The negative values are located in areas of lower accessibility (high altitudes and sharp slopes) and near to the watershed outlet (17.5% of the watershed area), whereas 82.5% of the area registered positive and, therefore, more reliable model outputs. It’s worth pointing out that the MESS method could be applied regardless of the chosen model, since all of three algorithms evaluated in this study offers the possibility for the selection of a subset of ‘most important covariates’, which could be of paramount importance to assess the map quality related to point-sampling, particularly when dealing with constrained sampling.

**Figure 8:**
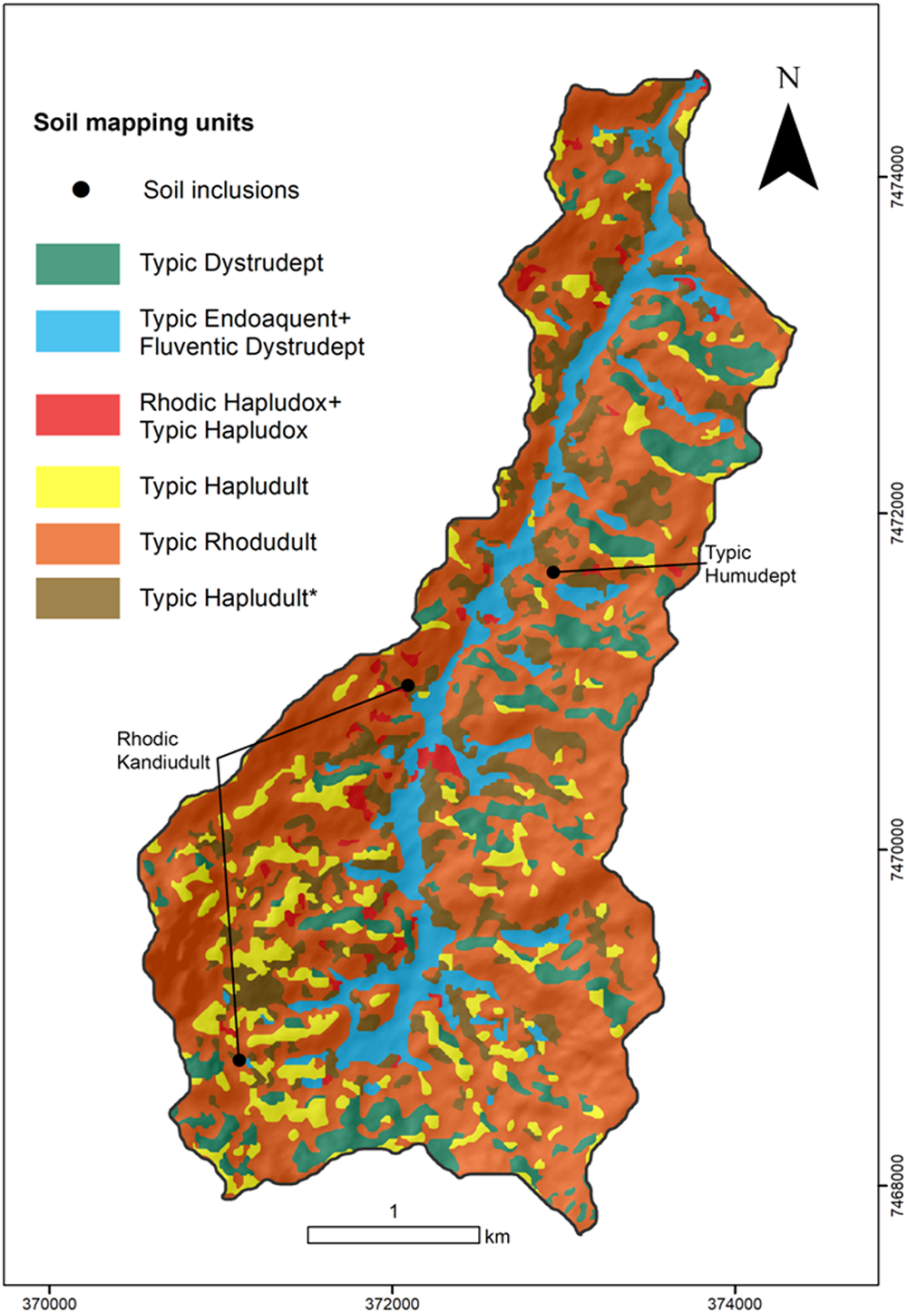
Soil mapping units distribution in the watershed by the RF* model.

**Figure 9:**
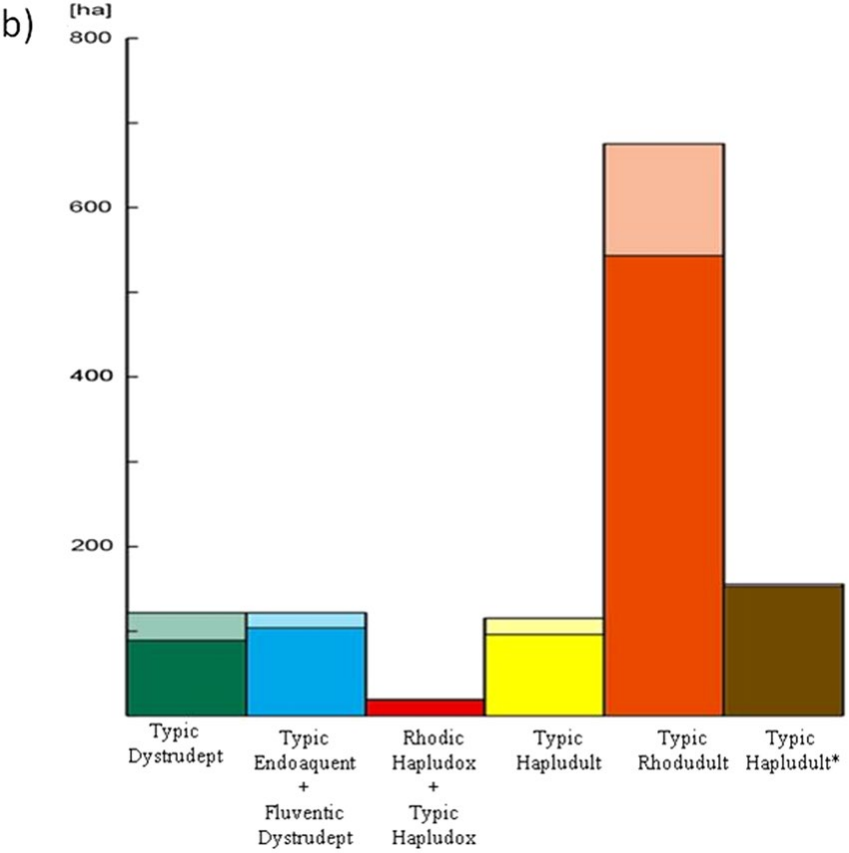
Extrapolation level of the digital soil map (lighter colors) by soil mapping unit.

The map showing the spatial distribution of the different soil mapping units predicted in the watershed is presented in Fig. 8. The majority of the soils in the Posses watershed were predicted as Typic Rhodudult, which proportion is in agreement with the distribution derived from the field measurements and additional point sampling. Figure 9 highlights the extrapolation level of each soil mapping unit. Typic Dystrudept (120 ha) and Typic Rhodudult (672 ha) mapping units have 27% and 20% of their areas in less reliable regions, respectively; while the other soil mapping units, with mapping areas of 120 ha - Typic Endoaquent + Fluventic Dystrudept, 156 ha - Typic Hapludult*, 108 ha - Typic Hapludult, and 24 ha - Rhodic Hapludox + Typic Hapludox, do not show an expressive uncertain area (<15%).

## Conclusions

A comparison among different models and the effects of additional point sampling for digital mapping of less-common soil classes, based on the spatial association with soilscape covariates, was performed. Tested models were Multinomial Logistic Regression, C5 Decision-Tree, and Random Forest. Additional point sampling retrieved by photointerpretation was necessary to improve the prediction performance of each model. The accuracy metrics were found to be higher for the Random Forest model with additional point sampling method, resulting in the best model. At the spatial resolution analyzed (20 m), the terrain-attribute covariates (relief data), related to surficial water distribution and direct insolation, were the most efficient to discriminate among soil mapping units and for producing a feasible digital soil map.

## Supplementary information

Supplementary Information

## References

[1] Brevik EC (2016). Soil mapping, classification, and pedologic modeling: History and future directions. Geoderma.

[2] Brevik EC, Hartemink AE (2010). Early soil knowledge and the birth and development of soil science. CATENA.

[3] Keys K, Sterling SM, Guan Y (2015). Using historic soil survey data to map water erosion hazard for land-use planning in Nova Scotia. Can. J. Soil Sci..

[4] Jenny, H. *Factors of Soil Formation: A System of Quantitative Pedology*. (McGraw-Hill, 1941).

[5] McBratney A, Mendonça Santos M, Minasny B (2003). On digital soil mapping. Geoderma.

[6] Miller BA, Schaetzl RJ (2016). History of soil geography in the context of scale. Geoderma.

[7] Shi R, Long R, Dekett R, Phillip R (2009). Integrating different types of knowledge for digital soil mapping. Soil Sci. Soc. Am. J..

[8] Zhu Q, Lin HS (2010). Comparing Ordinary Kriging and Regression Kriging for Soil Properties in Contrasting Landscapes. Pedosphere.

[9] Zhu, A. X. A similarity model for representing soil spatial information. **77**, 217–242 (1997).

[10] Bui EN (2004). Soil survey as a knowledge system. Geoderma.

[11] Hudson BD (1992). The Soil Survey as Paradigm-based Science. Soil Sci. Soc. Am. J..

[12] Godinho Silva SH, Owens PR, Duarte de Menezes M, Reis Santos WJ, Curi N (2014). A Technique for Low Cost Soil Mapping and Validation Using Expert Knowledge on a Watershed in Minas Gerais, Brazil. Soil Sci. Soc. Am. J..

[13] Cook SE, Corner RJ, Grealish G, Gessler PE, Chartres CJ (1996). A Rule-based System to Map Soil Properties. Soil Sci. Soc. Am. J..

[14] Camera C (2017). A high resolution map of soil types and physical properties for Cyprus: A digital soil mapping optimization. Geoderma.

[15] Jeune W (2018). Multinomial Logistic Regression and Random Forest Classifiers in Digital Mapping of Soil Classes in Western Haiti. Rev. Bras. Ciência do Solo.

[16] Taghizadeh-Mehrjardi R, Nabiollahi K, Minasny B, Triantafilis J (2015). Comparing data mining classifiers to predict spatial distribution of USDA-family soil groups in Baneh region, Iran. Geoderma.

[17] Brungard CW, Boettinger JL, Duniway MC, Wills SA, Edwards TC (2015). Machine learning for predicting soil classes in three semi-arid landscapes. Geoderma.

[18] Chawla NV, Bowyer KW, Hall LO, Kegelmeyer WP (2002). SMOTE: Synthetic Minority Over-sampling Technique. J. Artif. Intell. Res..

[19] Millard K, Richardson M (2015). On the Importance of Training Data Sample Selection in Random Forest Image Classification: A Case Study in Peatland Ecosystem Mapping. Remote Sens..

[20] Grunwald, S. In *Environmental soil-landscape modeling: Geographic information technologies and pedometrics* (ed. Grunwald, S.) 3–36 (Taylor & Francis, 2006).

[21] Minasny B, McBratney AB (2016). Digital soil mapping: A brief history and some lessons. Geoderma.

[22] Menezes MD, de, Silva SHG, Mello CR, de, Owens PR, Curi N (2014). Solum depth spatial prediction comparing conventional with knowledge-based digital soil mapping approaches. Sci. Agric..

[23] Meier M, Souza E, de, Francelino MR, Fernandes Filho EI, Schaefer CEGR (2018). Digital Soil Mapping Using Machine Learning Algorithms in a Tropical Mountainous Area. Rev. Bras. Ciência do Solo.

[24] Chagas CdaS (2017). Data mining methods applied to map soil units on tropical hillslopes in Rio de Janeiro, Brazil. Geoderma Reg..

[25] Giasson E, Clarke RT, Inda Junior AV, Merten GH, Tornquist CG (2006). Digital soil mapping using multiple logistic regression on terrain parameters in southern Brazil. Sci. Agric..

[26] Menezes MDD, Silva SHG, Owens PR, Curi N (2013). Digital soil mapping approach based on fuzzy logic and field expert knowledge. Ciência e Agrotecnologia.

[27] Pelegrino MHP (2016). Mapping soils in two watersheds using legacy data and extrapolation for similar surrounding areas. Ciência e Agrotecnologia.

[28] Alvares CA, Stape JL, Sentelhas PC, de Moraes Gonçalves JL, Sparovek G (2013). Köppen’s climate classification map for Brazil. Meteorol. Zeitschrift.

[29] Regional, C.-S. geológico do B. Mapa geológico do estado de Minas Gerais. at, http://rigeo.cprm.gov.br/jspui/handle/doc/5016 (2003)

[30] Pereira, P. H., Cortez, B. A., Trindade, T. & Mazochi, M. N. Conservador das Águas, 5 anos (2010).

[31] IBGE. *Manual Técnico de Pedologia*. (Coordenação de Recursos Naturais e Estudos Ambientais, 2015).

[32] GLINKA, K. D. & Marbut, C. F. The great soil groups of the world and their development. *Nat. Publ. Gr*. **126** (1927).

[33] Jenny HEW (1961). Hilgard and the Birth of Modern Soil Science. Soil Sci..

[34] Hengl T (2006). Finding the right pixel size. Comput. Geosci..

[35] Soil Survey Staff. *Keys to Soil Taxonomy*. (USDA-Natural Resources Conservation Service). at, https://www.nrcs.usda.gov/wps/PA_NRCSConsumption/download?cid=stelprdb1252094&ext=pdf (2014).

[36] dos Santos, H. G. *et al*. *Sistema Brasileiro de Classificação de Solos*. (Embrapa). at http://ainfo.cnptia.embrapa.br/digital/bitstream/item/181677/1/SiBCS-2018-ISBN-9788570358172.epub (2018).

[37] Hudson HD (1992). The Soil Survey as Paradigm-based Science. Soil Sci. Soc. Am. J..

[38] Corcoran J, Knight J, Gallant A (2013). Influence of Multi-Source and Multi-Temporal Remotely Sensed and Ancillary Data on the Accuracy of Random Forest Classification of Wetlands in Northern Minnesota. Remote Sens..

[39] Abbaszadeh Afshar F, Ayoubi S, Jafari A (2018). The extrapolation of soil great groups using multinomial logistic regression at regional scale in arid regions of Iran. Geoderma.

[40] Tadono T (2016). Generation of the 30 m-mesh global digital surface model by alos prism. Isprs - Int. Arch. Photogramm. Remote Sens. Spat. Inf. Sci..

[41] Rouse, J. W. J., Haas, R. H., Schell, J. A. & Deering, D. W. Monitoring vegetation systems in the Great Plains with ERTS. *In 3rd Earth Resource Technology Satellite (ERTS) Symposium* 48–62 (1974).

[42] Tucker CJ (1979). Red and photographic infrared linear combinations for monitoring vegetation. Remote Sens. Environ..

[43] Gao B-C (1996). NDWI—A normalized difference water index for remote sensing of vegetation liquid water from space. Remote Sens. Environ..

[44] Conrad, O. *et al*. System for Automated Geoscientific Analyses (SAGA) v. 2.1.4. *Geosci. Model Develoment* 1991–2007, 10.5194/gmd-8-1991-2015 (2015).

[45] R-Core-Team. R: A language and environment for statistical computing. at, www.R-project.org (2017).

[46] Kempen B, Brus DJ, Heuvelink GBM, Stoorvogel JJ (2009). Updating the 1:50,000 Dutch soil map using legacy soil data: A multinomial logistic regression approach. Geoderma.

[47] Lane PW (2002). Generalized linear models in soil science. Eur. J. Soil Sci..

[48] Campling P, Gobin A, Feyen J (2002). Logistic Modeling to Spatially Predict the Probability of Soil Drainage Classes. Soil Sci. Soc. Am. J..

[49] Strobl C, Malley J, Tutz G (2009). An introduction to recursive partitioning: Rationale, application, and characteristics of classification and regression trees, bagging, and random forests. Psychol. Methods.

[50] Quinlan, J. R. C4.5: *Programs for machine learning*. Morgan Kaufmann 5 (1993).

[51] Breiman L (2001). Random forests. Mach. Learn..

[52] Liaw A, Wiener M (2002). Classification and Regression by randomForest. R News.

[53] Kuhn, M. & Johnson, K. *Applied predictive modeling*. (Springer, 2013).

[54] Elith J, Kearney M, Phillips S (2010). The art of modelling range-shifting species. Methods Ecol. Evol..

[55] Zurell D, Elith J, Schröder B (2012). Predicting to new environments: tools for visualizing model behaviour and impacts on mapped distributions. Divers. Distrib..

[56] Barthold FK (2013). Land use and climate control the spatial distribution of soil types in the grasslands of Inner Mongolia. J. Arid Environ..

[57] Jafari A, Khademi H, Finke PA, Van de Wauw J, Ayoubi S (2014). Spatial prediction of soil great groups by boosted regression trees using a limited point dataset in an arid region, southeastern Iran. Geoderma.

[58] Hengl T (2017). Soil nutrient maps of Sub-Saharan Africa: assessment of soil nutrient content at 250 m spatial resolution using machine learning. Nutr. Cycl. Agroecosystems.

[59] Rossiter DG, Zeng R, Zhang G-L (2017). Accounting for taxonomic distance in accuracy assessment of soil class predictions. Geoderma.

[60] Heung B (2016). An overview and comparison of machine-learning techniques for classification purposes in digital soil mapping. Geoderma.

[61] Welling, S. H., Refsgaard, H. H. F., Brockhoff, P. B. & Clemmensen, L. H. Forest Floor Visualizations of Random Forests. (2016).

[62] Beven, K. & Kirkby, N. A physically based variable contributing area model of basin hydrology. *Hydrol. Sci. Bull*. 43–69 (1979).

[63] Bagheri Bodaghabadi M (2011). Using Canonical Correspondence Analysis (CCA) to identify the most important DEM attributes for digital soil mapping applications. CATENA.

[64] Machado IR (2018). Spatial Disaggregation of Multi-Component Soil Map Units Using Legacy Data and a Tree-Based Algorithm in Southern Brazil. Rev. Bras. Ciência do Solo.

[65] Nauman TW, Thompson JA (2014). Semi-automated disaggregation of conventional soil maps using knowledge driven data mining and classification trees. Geoderma.

[66] Silva S (2016). Proximal Sensing and Digital Terrain Models Applied to Digital Soil Mapping and Modeling of Brazilian Latosols (Oxisols). Remote Sens..

[67] Boehner, J. & Antonic, O. In *Geomorphometry - Concepts, Software, Applications* 195–226 (Elsevier, 2009).

[68] Chagas C, da S, Fernandes Filho EI, Bhering SB (2013). Relação entre atributos do terreno, material de origem e solos em uma área no noroeste do estado do Rio de Janeiro. Soc. Nat..

[69] Ferreira FP, Azevedo AC, Kanieski AJ, Girelli D, Pedrotti J (2005). Solar Exposure and Soil Properties in Santa Maria – RS. Rev. Bras. Agrociência.

