# Author Correction: Digital soil mapping including additional point sampling in Posses ecosystem services pilot watershed, southeastern Brazil

**DOI:** 10.1038/s41598-019-52011-0

**Published:** 2019-11-01

**Authors:** Bárbara Pereira Christofaro Silva, Marx Leandro Naves Silva, Fabio Arnaldo Pomar Avalos, Michele Duarte de Menezes, Nilton Curi

**Affiliations:** Departamento de Ciência do Solo, Universidade Federal de Lavras UFLA, Av. Doutor Sylvio Menicucci, 1001, Kennedy, Lavras, MG Brazil

Correction to: *Scientific Reports* 10.1038/s41598-019-50376-w, published online 24 September 2019

The Acknowledgements section in this Article is incomplete.

“This research was funded by Coordination of Superior Level Staff Improvement – CAPES – Finance Code 001, National Council of Technological and Scientific Development – CNPq (Process n^o^306511/2017-7 e 202938/2018-2) and Minas Gerais State Research Foundation – FAPEMIG (Process n^o^APQ-00802-18 and CAG-APQ-01053-15).”

should read:

“This research was funded by Coordination of Superior Level Staff Improvement – CAPES – Finance Code 001, CAPES/PROEX/AUXPE 593/2018,National Council of Technological and Scientific Development – CNPq (Process n^o^306511/2017-7 e 202938/2018-2) and Minas Gerais State Research Foundation – FAPEMIG (Process n^o^APQ-00802-18 and CAG-APQ-01053-15).”

