# Supplementary material for: Digital soil mapping including additional point sampling in Posses ecosystem services pilot watershed, southeastern Brazil
Source: Sci Rep. 2019 Sep 24;9:13763. doi: 10.1038/s41598-019-50376-w (PMC6760152; doi:10.1038/s41598-019-50376-w)

Digital soil mapping including additional point sampling in Posses ecosystem services pilot watershed, southeastern Brazil

Bárbara Pereira Christofaro Silva*, Marx Leandro Naves Silva, Fabio Arnaldo Pomar Avalos, Michele Duarte de Menezes, Nilton Curi

Supplementary Table S1: Evaluated covariates for the digital soil mapping process

SCORPAN factor	Evaluated covariate	Spatial resolution[1] (m)
Organisms and vegetation	Sentinel 2 Band 2	10
	Sentinel 2 Band 3	10
	Sentinel 2 Band 4	10
	Sentinel 2 Band 8	10
	Sentinel 2 Band 5	20
	Sentinel 2 Band 6	20
	Sentinel 2 Band 7	20
	Sentinel 2 Band 8a	20
	Sentinel 2 Band 11	20
	Sentinel 2 Band 12	20
	Sentinel 2 Band 1	60
	Sentinel 2 Band 9	60
	Sentinel Band 2 10	60
	Normalized Difference Vegetation Index	20
	Normalized Difference Water Index	20
Topography	Elevation	12.5
	Cross-Sectional Curvature	20
	Diffuse Insolation	20
	Direct Insolation	20
	Diurnal Anisotropic Heating	20
	Downslope Curvature	20
	Flow Line Curvature	20
	General Curvature	20
	Local Curvature	20
	Local Downslope Curvature	20
	Local Upslope Curvature	20
	Longitudinal Curvature	20
	Maximal Curvature	20
	Minimal Curvature	20
	Multiresolution Ridge Top Flatness	20
	Multiresolution Index of Valley Bottom Flatness	20
	Negative Openness	20
	Plan Curvature	20
	Positive Openness	20
	Profile Curvature	20
	Slope	20
	Stream Power Index	20
	Tangential Curvature	20
	Terrain View Factor	20
	Topographic Wetness Index	20
	Total Curvature	20
	Total Insolation	20
	Upslope Curvature	20

*The covariates with spatial resolutions less than the mapping resolution (20 m) were upscaled, and those with higher resolution were downscaled, both by bilinear interpolation

Digital soil mapping including additional point sampling in Posses ecosystem services pilot watershed, southeastern Brazil

Bárbara Pereira Christofaro Silva*, Marx Leandro Naves Silva, Fabio Arnaldo Pomar Avalos, Michele Duarte de Menezes, Nilton Curi

Supplementary Figure S2. Spearman rank correlation matrix between the most important covariates in the Random Forest with additional observations model.

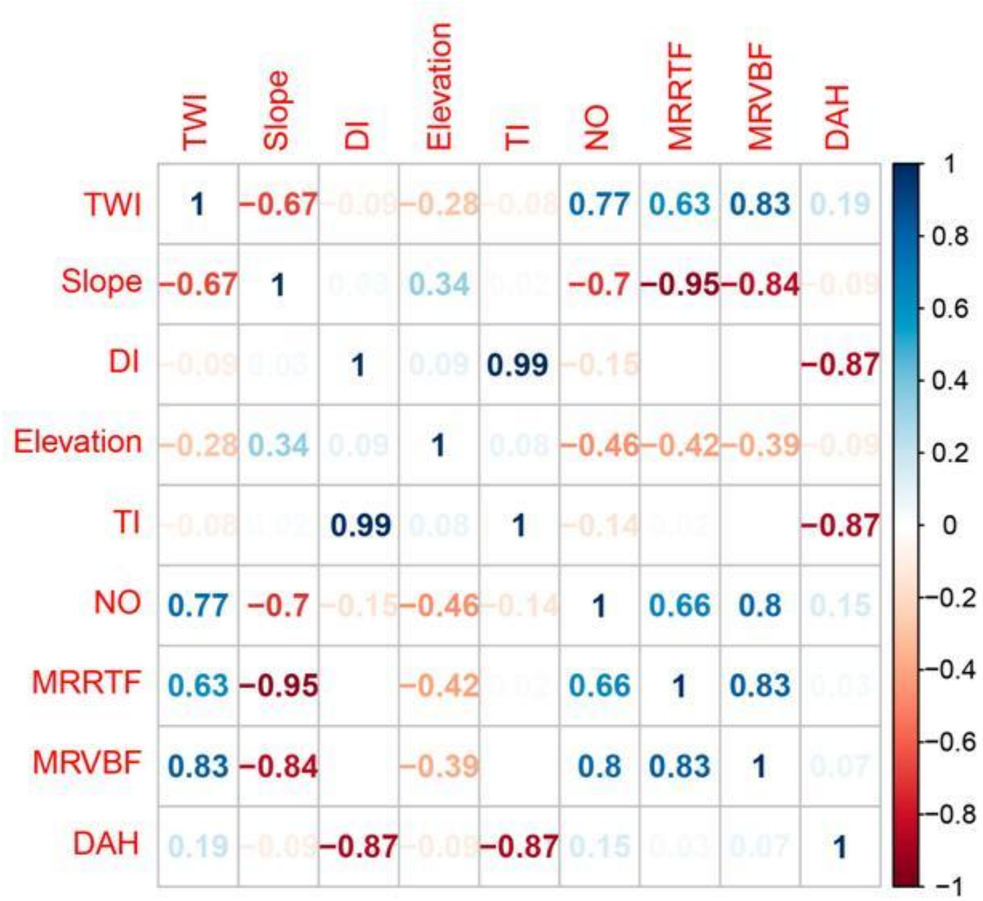

TWI: Topographic Wetness Index, DI: Direct Insolation, TI: Total Insolation, NO: Negative Openness, MRRTF: Multiresolution Ridge Top Flatnes, MRVBF: Multiresolution Index of Valley Bottom, DAH: Diurnal Anisotropic Heating

Supplementary Figure S3. Covariate importance rank measured by the mean decrease in accuracy in the Random Forest with additional observations model from which the most important covariates were selected

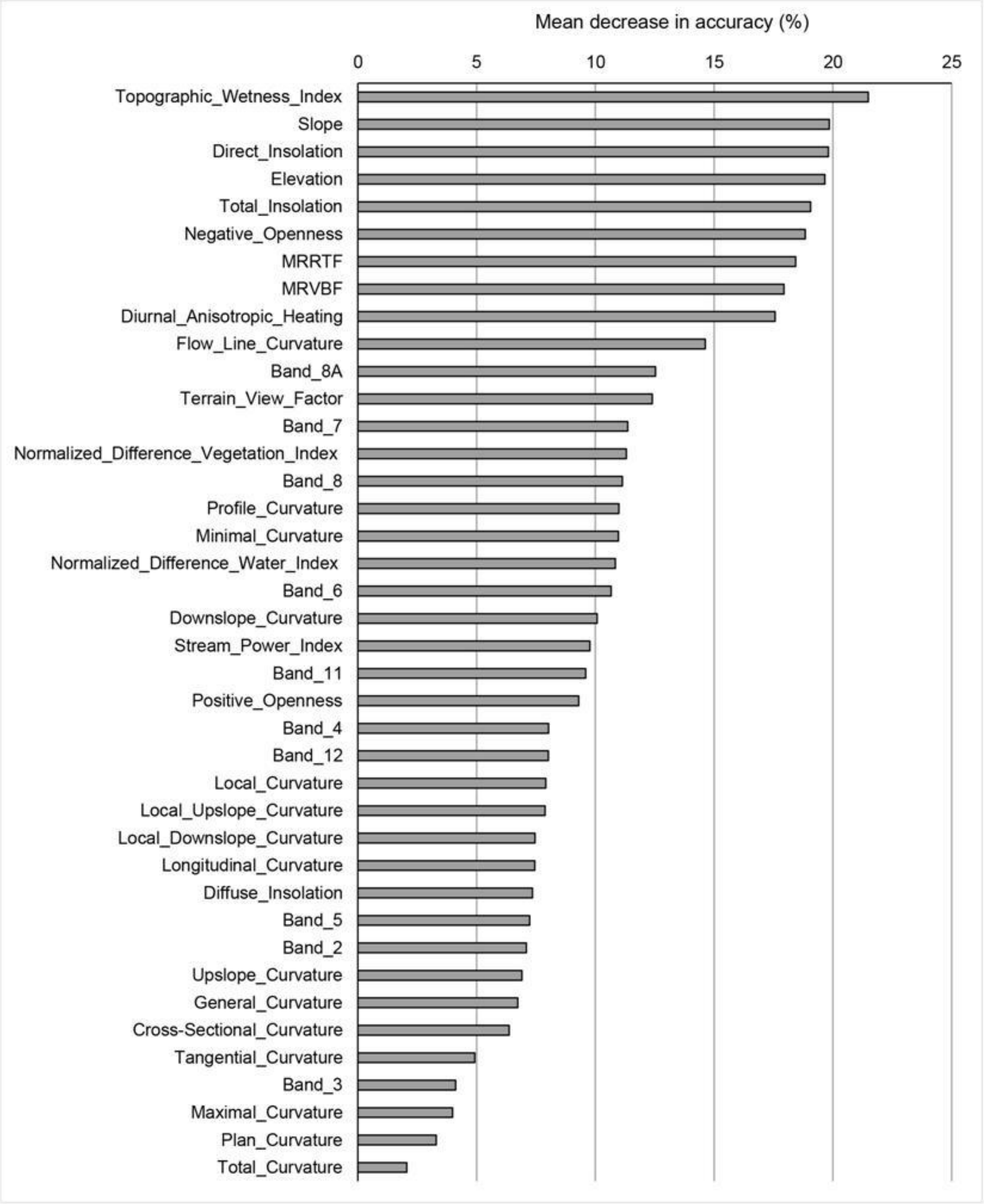

Supplement: Supplementary file 1 — Supplementary Information [file 41598_2019_50376_MOESM1_ESM.pdf]
